# Supplementary material for: Bartonella- and Borrelia-Related Disease Presenting as a Neurological Condition Revealing the Need for Better Diagnostics
Source: Microorganisms. 2024 Jan 19;12(1):209. doi: 10.3390/microorganisms12010209 (PMC10819350; doi:10.3390/microorganisms12010209)
Supplement: Supplementary file 1 [file microorganisms-12-00209-s001.zip › microorganisms-2812565-supplementary.pdf]

Table S1: Sequence confirmation that primers amplify correct target.

Gene Name	Direct Sequencing of PCR amplicon using Hemi-nested or Touch Down PCR	GenBank Accession number
Bartonella vinsonii subsp. berkhoffii 16S ribosomal RNA gene, partial sequence (16S-23S ribosomal RNA intergenic spacer)	GAGGACTTGAACCTCCGACCTCACGCTTATCAAG<br>CGCGCGCTCTAACCAACTGAGCTACAAGCCCTCC<br>GGGATAAACCGGAAAACCTTCCCCGGAAAACTC<br>TTAAAAAAGCAAACTCTCAAATAAAAGAGCAAA<br>CTCTTAGATAAAAAATACACTCTTACAGGAAAGT<br>TTTTCTTTAATTTTATAAAAAACTTTTTCTTAAAGG<br>GTGTTTTCTTAAAAAGCCCTCTTTTTT	AF312503.1
Bartonella henselae strain Houston-I chromosome, complete genome (16S-23S ribosomal RNA intergenic spacer)	GAGGACTTGAACCTCCGACCTCACGCTTATCAAG<br>CGCGCGCTCTAACCAACTGAGCTACAAGCCCTCC<br>GGGATAAACCGGAAAACCTTCCCGGGCATCCTCT<br>TATAAAACGCTTAAATTTTATCAAGGAAAGCGTTT<br>TTCAAAGCCCACGGTGGACTTGTTTTGTCTAGATC<br>GCCAGAAGGCTTGGGATCATCATCTGAAG	CP020742.1
Bartonella henselae strain Houston-I chromosome, complete genome (BRT1)	CCTGGAAGCTCTAACATCGAACACAGAATAAATC<br>CGCCTTGCGGGTATCCCGGAATTTCCGGGGGCTCT<br>TGTTATGTCCAAGTGCTTCTCAGCACTAAAAGCA<br>AGAGACTGATTCGATGTCAGTGCTTCCAGACC	CP020742.1
Borreliella burgdorferi isolate Chosun M10-8Sp outer surface protein A (OspA) gene, partial cds	TTCAAGTGTGGTTTGACCTAGATCGTCAGAAATTG<br>TTAATTTTACTTTACTTTTGTCAGCTTCTACGCCTT<br>CAAGTACTCCAGATCCATTGTTTTTATCAGAAGTT<br>CCTTTAAGCTCAAGCTTGTCTACTGTTGCAATTAG<br>ATCGTACTTGCCGTCTTTGTTTTTTTCTTTGCTTAC<br>AAGAACTTTCATTTCACCAGGCAAGTCTACTGAA<br>ACGCTGTTT	MF948162.1
Borreliella burgdorferi strain MM1 chromosome main, complete sequence (P13)	ATGAAACTAGCAAGCAAGATCCTATTGTACCATTT<br>TTATTGAACCCTTTTTTAGGGTTTGGAATAGGCTC<br>CTTTGCTCAAGGAGATATTCTTGGAGGTTCTCTTA<br>TTCTTGGATTTGATGCGGTTGGTATAGGGCA	CP031412.1

Babesia microti isolate GXS54 small subunit ribosomal RNA gene, partial sequence (18S)	GAAGACGATCAGATACCGTCGTAGTCCTAACCATAAACTATGCCGACTAGAGATTGGAGGTCGTCAGTTTAAACGACTCCTTCAGCACCTTGAGAGAAATCAAAGTCTTTGGGTTCTGGGGGGAGTATGGTCGCAAGTCCGAAACTTAAAGGAATTGACGGAAGGGCACCA	OR117721.1
